# Tissue Adaptations of Memory and Tissue-Resident Gamma Delta T Cells

**DOI:** 10.3389/fimmu.2018.02636

**Published:** 2018-11-27

**Authors:** Camille Khairallah, Timothy H. Chu, Brian S. Sheridan

**Affiliations:** Department of Molecular Genetics and Microbiology, Center for Infectious Diseases, Stony Brook University, Stony Brook, NY, United States

**Keywords:** memory γδ T cells, resident γδ T cells, innate γδ T cells, adaptive γδ T cells, barrier infections

## Abstract

Epithelial and mucosal barriers are critical interfaces physically separating the body from the outside environment and are the tissues most exposed to microorganisms and potential inflammatory agents. The integrity of these tissues requires fine tuning of the local immune system to enable the efficient elimination of invasive pathogens while simultaneously preserving a beneficial relationship with commensal organisms and preventing autoimmunity. Although they only represent a small fraction of circulating and lymphoid T cells, γδ T cells form a substantial population at barrier sites and even outnumber conventional αβ T cells in some tissues. After their egress from the thymus, several γδ T cell subsets naturally establish residency in predetermined mucosal and epithelial locations, as exemplified by the restricted location of murine Vγ5^+^ and Vγ3Vδ1^+^ T cell subsets to the intestinal epithelium and epidermis, respectively. Because of their preferential location in barrier sites, γδ T cells are often directly or indirectly influenced by the microbiota or the pathogens that invade these sites. More recently, a growing body of studies have shown that γδ T cells form long-lived memory populations upon local inflammation or bacterial infection, some of which permanently populate the affected tissues after pathogen clearance or resolution of inflammation. Natural and induced resident γδ T cells have been implicated in many beneficial processes such as tissue homeostasis and pathogen control, but their presence may also exacerbate local inflammation under certain circumstances. Further understanding of the biology and role of these unconventional resident T cells in homeostasis and disease may shed light on potentially novel vaccines and therapies.

## Introduction

Epithelial and mucosal tissues form physical barriers separating the body from the outside world. They are constantly exposed to a wide range of stressors such as infectious agents and their toxins capable of damaging barrier tissues. Barrier surface interactions with microorganisms extend far beyond encounters with pathogenic microbes; indeed, these tissues are typically mutualistic ecosystems that maintain beneficial relationships for resident commensal organisms while providing support to the tissue (1). Because of the complexity of these interfaces, the immune system is tightly regulated in order to eliminate invading pathogens while maintaining a robust commensal environment. It is now well established that the microbiota plays a significant role in educating immune cells and promoting protective anti-infectious responses (2–4). However, the microbiota may also play an important role in aberrant inflammation (5, 6). In addition, pathogenic agents also leave their imprint on the immune system and generate long-lasting memory responses. Protective immunity has mainly been the purview of conventional effector memory (T_EM_) or central memory (T_CM_) T cells and B cells. More recently, the discovery of conventional resident memory T cells (T_RM_) (7, 8), innate immune memory also known as trained immunity (9, 10), and other unconventional memory responses (11, 12) has focused attention on tissue-specific immunity at barrier locations.

γδ T cells are an unconventional T cell population that display immunologic features common to both the innate and adaptive immune systems (13). This dual nature of γδ T cell biology is typified by their non-MHC-restricted antigenic specificity while mounting rapid immune responses to a wide range of tissue stressors (14), generally referred to as “lymphoid-stress surveillance” (15). γδ T cells are the first T cells generated during embryonic development and quickly seed peripheral tissues where specialized subsets are maintained for life in residence. These unconventional T cells are only found at low frequencies in lymphoid tissues and the blood in adult humans and rodents; however, they are enriched in epithelial and mucosal tissues (16–19). Generally, distinct barrier tissues harbor mostly non-overlapping γδ T cell subsets with non-redundant functions (17). Some tissues contain unique and highly specialized γδ T cell subsets that are not found elsewhere in the body. For example, Vγ3Vδ1^+^ skin dendritic epidermal T cells (DETC) reside exclusively in the skin epidermis while Vγ5^+^ T cells reside exclusively in the intestinal epithelium [the Garman nomenclature (20) is used throughout this review for murine γδ T cells] (21). The development and selection processes that regulate the differentiation of these cells are unique and result in the generation of highly adapted cells that actively survey neighboring cells, sense and respond to stresses of various nature and participate in many tissue processes. Thus, these natural tissue-resident γδ T cells are programmed sentinels that are also shaped by and highly adapted to their tissue environment.

Because of their preferential location in barrier sites, γδ T cells are often directly or indirectly influenced by the microbiota or the pathogens that invade these sites. The steady-state microbiota may influence the generation, effector functions, or maintenance of γδ T cells (22–24). These commensal-induced γδ T cells adapt to their tissue of residence where they add another level of immune surveillance and may be mobilized in many pathological contexts including inflammation (25–27) and cancer (28, 29). These tissue-resident γδ T cells are also mobilized during infection to promote anti-pathogen immunity (30) and represent innate first responders during infection. Alternatively, pathogen-induced adaptive γδ T cells appear to follow a more conventional T cell maturation pathway, resulting in delayed activation and expansion while favoring the establishment of long-lasting memory and heightened protective potential upon pathogen re-exposure. Throughout this review, the term “adaptive” will be utilized to describe γδ T cells having features consistent with conventional αβ T cells. This review will focus on the tissue adaptation of tissue-resident natural γδ T cells and adaptive γδ T_RM_ cells in barrier tissues while highlighting their development, maintenance and role in health and disease.

## γδ T cells of humans and mice

Murine γδ T cells are often segregated into different subsets based on their Vγ T cell receptor (TCR) chain, as it is generally associated with tissue tropism and a bias in effector function (31, 32) (Table 1). It is well established that γδ T cell ontogeny is temporally controlled and manifested by “waves” of development (76). The factors regulating γδ T cell development have been recently reviewed (77, 78). Most barrier tissue γδ T cells develop early during fetal development in the fetal/neonatal thymus with the first thymic wave of γδ T cells starting at embryonic day 13 and giving rise to DETC characterized by surface expression of an invariant Vγ3Vδ1 TCR (16). Vγ3Vδ1^+^ DETC migrate to the skin epidermis (18, 76, 79) and produce IFNγ (80) and other cytokines (81, 82), and growth factors (83, 84). From embryonic day 14 to the perinatal period, the fetal/neonatal thymus generates other innate-like [also called “natural” (85)] γδ T cells, including the IL-17A biased quasi-invariant Vγ4Vδ1^+^ T cells which preferentially migrate to the genital tract, the tongue and the lungs (16, 76, 86). Fetal-derived γδ T cells are typically considered innate-like due to their reduced TCR sensitivity (87) and rapid functional response to innate stimuli like cytokines and pathogen- or danger-associated molecular patterns (72, 88, 89). IL-17A-producing γδ T cells (referred to as γδ17 T cells in this review) are characterized by the expression of the transcription factor RORγt (90), chemokine receptor CCR6 (86, 90, 91), scavenger receptor SCART2 (92), CD25 (93), but lack CD27 (86, 90, 94). In contrast, IFNγ-producing γδ T cells express the transcription factor T-bet and surface receptors NK1.1 and CD27. Consistent with other IFNγ producing lymphocytes, they also express high levels of the IL-2/IL-15 receptor β chain CD122 (93, 95). It was initially thought that γδ17 T cells acquired their peripheral effector fate due to a lack of antigenic selection in the thymus; antigen-experienced cells were programmed to make IFNγ in the periphery while antigen-inexperienced cells were programmed to make IL-17A (80, 87, 95, 96). However, recent evidence suggests that signaling through the TCR is required for γδ17 T cells development and that the strength of the signal is the critical factor determining their functional lineage. A strong TCR signal promotes an IFNγ-dominant lineage whereas a weak TCR signal promotes an IL-17A-dominant lineage (97–99). An additional level of regulation comes from the thymic cytokine milieu: while signaling through IL-15Rα restrains γδ17 T cell development *in cis* (100), IL-7 promotes their expansion (101). An interesting feature of γδ17 T cells is their functional plasticity, which allows them to co-produce IL-17A and IFNγ under certain circumstances (61, 102). Although CD27^−^ γδ T cells have a permissive chromatin state at the *Il17a* and *Ifng* loci, only a handful of situations have been associated with IL-17A and IFNγ co-production *in vivo*, including oral *Listeria monocytogenes* (*L. monocytogenes*) infection (61, 62) and peritoneal tumor (102). Post-transcriptional repression of IFNγ production has recently been reported in γδ17 T cells (61); however, whether co-production of IL-17A and IFNγ is regulated by derepression has not been evaluated.

**Table 1:** Memory and tissue resident γδ T cells in infection and disease.

**Tissue**	**Subset(s)**	**Role**	**Response**	**Cytokines**	**Other features**	**Context**	**References**
Systemic	Vδ2^−^	Protective	Memory?	IFNγ	Stress surveillance against CMV and cancer	CMV	(33–37)
	ND	Protective	Memory?	IFNγ	Antigen specific expansion	Vaccinia	(38)
	Vγ9Vδ2	Protective	Memory	IFNγ	Cross-reactive to HMBPP	Monkeypox	(39)
	Vγ9Vδ2, Vδ1	Protective	Memory?	IFNγ	Late expansion after initial exposure	*P. falciparum*	(40–43)
	Vγ1.1Vδ6.3	Protective	Memory?	M-CSF, CCL5, CCL3	Oligoclonal expansion	*P. chabaudi*	(44)
	Vγ9Vδ2	Protective	Memory	IFNγ	Cross-react with *M. tuberculosis*	BCG	(45, 46)
Lungs	Vγ9Vδ2	Protective	Memory	Granzyme B	Activated by HMBPP	*M. tuberculosis*	(45–47)
	Vγ1.1^−^, Vγ2^−^	Protective	Innate	IL-17A	High expression of IL-1R1, IL-18R, and IL-23R	*B. pertussis*	(48)
	Vγ2	Protective	RM	IL-17A	*B. pertussis*-specific	*B. pertussis*	(48)
Peritoneum	Vγ4	Protective	RM	IL-17A	CD27-CD44+ Effector memory phenotype	*S. aureus*	(49)
	Vγ1.1, Vγ2	Protective	Innate	ND	Polyclonal response	*S. aureus*	(49)
Skin	Vγ4Vδ1	Protective	RM	IFNγ, TNFα	TLR2/IL-1β dependent response	*S. aureus*	(50)
	Vγ2Vδ4	Pathogenic	RM	IL-17A/F	CCR2-dependent recruitment to tissue	Psoriasis	(25, 26)
	Vγ2	Pathogenic	RM	IL-17A	Constitutive expression of CCR6, RORγt, and IL-23R	Dermatitis	(51)
	Vγ9Vδ2, Vδ1	Variable	Memory	IL-17A, IFNγ, TNFα	Pathogenic IL-17A; Protective IFNγ	SCC/Melanoma	(52, 53)
	Vγ3Vδ1	Protective	Innate	IFNγ, KGF-1/2	Immotile; semi-activated	Wound, dermatitis, *S. aureus*, cancer	(54–58)
Intestine	Vγ9Vδ2	Protective	Memory	IL-17A, IFNγ, IL-4, TNFα	Multifunctional cytokine production	*L. monocytogenes*	(59, 60)
	Vγ4Vδ1	Protective	RM	IL-17A, IFNγ	Multifunctional cytokine production	*L. monocytogenes*	(61, 62)
	Vδ1	Pathogenic	Infiltrating	IFNγ	Interacts with colonic fibroblasts	IBD	(63, 64)
	Vγ9Vδ2, Vδ1	Pathogenic	RM	GM-CSF, IL-17A	Pathogenicity dependent on MDSC regulation	CRC	(65)
	Vγ5, others	Protective	Innate	IFNγ, Granzymes	Highly motile; semi-activated	*S. enterica, T. gondii*	(66–69)
Breast	Vγ2	Pathogenic	RM	G-CSF, IL-17A	Pathogenicity dependent on MDSC regulation	Breast Cancer	(70)
Brain	Vδ2, Vδ1	Protective	Memory	IFNγ, TNFα, Granzyme B	Found in the context of γδ expansion methodology	Neuroblastoma	(71)
	Vγ2	Pathogenic	Innate	IL-17 cytokines, IL-21	IL-23- and IL-1β-dependent activation	EAE/MS	(72)
	ND	Pathogenic	Innate	IL-17A	Part of a microbiota-gut-brain axis	Ischemic stoke	(27)
Joints	Vγ1.1, Vγ1.2	Pathogenic	Innate	IL-17A	IL-23- and IL-1β-dependent activation	CIA	(73)
	ND	Pathogenic	Innate	IL-17A	IL-23-dependent activation	Ankylosing spondylitis	(74)
Eyes	Vγ1.1, Vγ2	Pathogenic	Innate?	IL-17A	Enhanced uveitogenic αβ T cell development	Uveitis/EAU	(75)
	Vy2	Protective	Resident	IL-17A	Induced by *C. mastidis* colonization	Ocular *P. aeruginosa*/	(24)
					CD1d- and IL-1β-dependent	*Candida albicans*	
Primate γδ T cells		Rodent γδ T cells					

*ND, not determined; RM, resident memory; CMV, cytomegalovirus; BCG, M. bovis BCG strain; SCC, squamous cell carcinoma; IBD, inflammatory bowel disease; CRC, colorectal cancer; EAE/MS, experimental autoimmune encephalomyelitis/multiple sclerosis; CIA, collagen-induced arthritis; EAU, experimental autoimmune uveitis*.

Although most γδ17 T cells fall into the innate-like category, adaptive-like differentiation of naïve γδ T cell precursors into mature γδ17 T cells in peripheral lymphoid organs has also recently been reported in multiple models. After the identification of phycoerythrin (PE) as a γδTCR antigen, PE-specific γδ T cells were shown to transition from a naïve CD44^lo^ CD62L^hi^ to an activated CD44^hi^ CD62L^lo^ phenotype after immunization with PE (103). These γδ T cells expressed RORγt and inflammatory cytokine receptors IL-1R1 and IL-23R which drove production of IL-17A without extensive proliferation (103). Similarly, imiquimod (IMQ)-induced skin inflammation and MOG-induced experimental autoimmune encephalomyelitis (EAE) induced the *de novo* generation of γδ17 T cells in draining lymph nodes (104, 105). These unrelated models demonstrate that the differentiation of some γδ17 T cell subsets is optimal with a TCR signal and in the presence of IL-23, reminiscent of the multistep development of naïve CD4^+^ T cells. In contrast to natural γδ17 T cells, these *de novo* generated cells are often referred to as inducible γδ17 T cells (14).

γδ T cell subsets in human and non-human primates are generally divided into two major populations based on the Vδ TCR chain: Vδ2^+^ and Vδ2^−^ γδ T cells. Vδ2^+^ T cells appear to develop almost exclusively in the fetal liver and fetal thymus (106, 107) and form the predominant γδ T cell population in the peripheral blood of adult humans (108, 109). Most fetal, cord blood and adult Vδ2^+^ T cells express the semi-invariant Vγ9Vδ2 TCR with a public germline encoded CDR3γ sequence and a more diverse CDR3δ sequence (110). Despite their preferential localization in the blood, Vγ9Vδ2^+^ T cells can also be recruited to inflamed tissues where they can participate in pathogen clearance or promote inflammation (39, 45, 47) (Table 1). The TCR combination allows the majority of Vγ9Vδ2^+^ T cells to recognize prenyl pyrophosphate metabolites (111), broadly referred to as phosphoantigens (PAgs), presented in the context of butyrophilin (BTN)3A1 and BTN3A2 (112–115). PAgs are metabolic intermediates produced by the eukaryotic mevalonate pathway and the microbial 2-C-methyl-D-erythriol 4-phosphate (MEP) pathway, which generates one of the most potent Vγ9Vδ2^+^ T cell activator (E)-4-hydroxy-3-methyl-but-2-enyl pyrophosphate (HMBPP) (111). Fetal Vγ9Vδ2^+^ T cells express genes found in adult cells and can expand and produce IFNγ in response to HMBPP stimulation (110). By 1 year of age, almost all Vγ9Vδ2^+^ T cells have acquired a memory phenotype and can rapidly produce IFNγ and cytotoxic molecules (108, 116), similar to circulating adult cells (108, 116, 117). These data suggest that human Vγ9Vδ2^+^ T cells are preprogrammed fetal-derived effectors with a restricted TCR specificity. Thus, Vγ9Vδ2^+^ T cells seem to belong to the natural, innate-like population of lymphocytes.

In contrast to Vγ9Vδ2^+^ T cells, the Vδ2^−^ γδ T cell subset is heterogenous (106) and preferentially resides in epithelial tissues such as the skin (118) and intestines (119) and appears to form resident populations in the liver (120) (Table 1). Vδ2^−^ γδ T cells mainly consist of Vδ1^+^ T cells, with fewer Vδ3^+^ and Vδ5^+^ T cells. While most antigens recognized by Vδ2^−^ γδ T cells remain unknown, the antigens identified to date suggest a broad reactivity to MHC-like molecules like endothelial protein C receptor (EPCR) (33) and CD1 molecules (33, 121, 122), stress-induced ligands (123) and algal phycoerythrin (103). Vδ2^−^ γδ T cell TCR are highly diverse in cord blood but their TCR repertoire becomes more restricted into adulthood (124). Furthermore, they clonally expand in response to cytomegalovirus (CMV) infection and differentiate into CD45RA^+^ effector memory T (T_EMRA_) cells (34, 35, 125–127). Thus, the Vδ2^−^ γδ T cell repertoire appears to be shaped by TCR-dependent selection events mediated by microbial encounters throughout life. As Vδ2^−^ γδ T cells can recognize stress antigens, non-infectious events that trigger a response, such as cancer development, may also shape their repertoire (36, 128).

γδ T cells can provide different physiologic roles depending on the nature and context of the insult, the tissue involved and the γδ T cell populations mobilized. At steady state, γδ T cells are involved in many biological processes aiming at maintaining barrier integrity (e.g., by promoting epithelial cell survival and homeostasis) (82–84, 129) and regulating thermogenesis (130). Because of their rapid sensing of stress and recruitment to inflamed sites, γδ T cells are often involved in shaping early immunologic events. They can promote the activation, maturation, and recruitment of dendritic cells (DC), neutrophils, B cells, and conventional T cells [for a detailed review see (131)]. γδ T cells are also a direct and potent source of critical inflammatory cytokines like IFNγ, TNFα and IL-17A in many pathological contexts, including infection (59, 111, 132–134), autoimmune disease (25, 26, 72, 135) and cancer (29, 136–138). As such, they are also an integral part of the effector response. At later phases, γδ T cells can promote the resolution of the inflammation through the production of anti-inflammatory molecules like TGFβ (139, 140). Finally, they sustain tissue repair and remodeling after infection or injury (54, 83, 132, 141). Thus, γδ T cells are critically involved in regulating health during homeostasis and disease.

## The first tissue-resident T cells: intestinal and epidermal γδ T cells

Many γδ T cell subsets are constrained to specific tissue locations. DETC and intestinal intraepithelial lymphocytes (IEL) with a γδTCR (γδ IEL) populate the two largest interfaces of the body, the skin and the intestines, respectively. DETC and γδ IEL are shaped within their respective tissues where they provide adapted support to maintain tissue homeostasis and respond to stresses or invading pathogens. These populations have recently been the focus of an in-depth review (21). Thus, only features relevant to this review will be discussed here.

### Dendritic epidermal T cells– DETC

DETC are the first T cells to develop during embryogenesis and by far the most abundant T cell subset present in the mouse skin epidermis (142). Their name stems from the unique DC-like morphology observed during homeostasis. DETC form a highly uniform population characterized by the expression of a canonical Vγ3Vδ1 TCR with no junctional diversity. The mouse fetal thymus supports the generation of the entire DETC precursor pool between embryonic day 13 and 18, after which mature DETC are maintained life-long in the skin epidermis by self-renewal (18, 76, 79, 143). The narrow developmental window of DETC progenitors may result from the temporally restricted expression of a Btn-like protein, Skint-1, by embryonic medullary thymic epithelial cells (144–146). Expression of Skint-1 is required at various stages of DETC thymic development to regulate their biology. First, Skint-1 promotes the thymic maturation of Vγ3Vδ1^+^ T cell progenitors, without which the skin epidermis would be devoid of mature DETC (144–146). Second, Skint-1 educates DETC precursors by promoting IFNγ production over IL-17A (80), instructing skin-homing (147), and attenuating TCR responsiveness by increasing its activation threshold (87). Similarly, TCR signaling seems required for the maturation of DETC precursors (148–150) and the establishment of a mature population with innate-like properties in the skin epidermis (87, 148–151). It is also indirectly involved in the thymic egress and subsequent migration to the skin of positively selected progenitor cells. Indeed, TCR signaling induced the expression of sphingosine-1-phosphate receptor 1 (S1P1) and the skin-homing chemokine receptor CCR10, which mediates T cell exit from the thymus and migration toward keratinocyte-derived CCL27, respectively (152, 153). Additional molecules like E- and P-selectin ligands and CCR4 may also play a role in the establishment or maintenance of the DETC population in the skin (154).

During homeostasis, mature DETC are maintained in a semi-activated state and constantly survey the epidermis through the extension of motile basal dendrites and by projecting dendrites toward the apical epidermis. These dendrites establish stable synapses at the squamous keratinocyte junctions that allows DETC to survey several surrounding cells simultaneously (155). Each apical dendrite ends with phosphorylated tyrosine–rich aggregates in synapse-like structures enriched with TCR and phosphorylated TCR signaling intermediates. Therefore, mature DETC might receive continuous TCR-mediated signals from neighboring cells residing in the epidermis, which are necessary for their long-term maintenance in the tissue (156). Although healthy skin does not appear to express DETC TCR ligand detectable by soluble Vγ3Vδ1 TCR tetramers (157), exposure of the skin to low grade stresses might sustain basal expression of ligands sufficient for their survival but below the sensitivity of this detection method. Indeed, DETC express basal levels of the type-2 cytokine IL-13 in resting skin, consistent with some level of activation at steady state (82). Absence of DETC-derived IL-13 induces an epithelial cell stress response that disrupts barrier integrity. As such, DETC play a key role in preserving skin homeostasis at steady state.

The skin is constantly exposed to a variety of pathological conditions and stresses. Superficial damage to the epithelium induces a stress response associated with upregulation of the NKG2D ligand Rae-1 and leads to the further activation of DETC (82, 158). Enhanced production of DETC-derived IL-13 induces keratinocyte maturation, which promotes efficient epithelial cell renewal, restoring tissue integrity (82). Shortly after deep wounding, damaged keratinocytes in close proximity to the lesion quickly and transiently upregulate a yet unidentified stress antigen (156, 157, 159). DETC rapidly become activated in a TCR dependent-manner and their activation is associated with retraction of their dendrites and cellular rounding (54, 155, 159). Full activation of DETC in this context requires engagement of the TCR and costimulation provided by the junctional adhesion molecule JAML (81), CD100 (semaphorin-4D) (160) or NKG2D (161, 162), whose ligands are all upregulated in damaged skin. Activated DETC provide anti-apoptotic signals to keratinocytes and promote their survival through the production of insulin-like growth factor-1 (84). DETC also produce many additional growth factors, including keratinocyte growth factor (KGF)-1 and KGF-2 (54, 83), inflammatory cytokines like IFNγ and TNFα (81, 163) and chemokines (164) that favor epithelial regeneration and wound closure. The important and non-redundant contribution of DETC to wound repair was demonstrated in *Tcrd*^−/−^ mice or animals deficient in DETC costimulatory signals. Lack of DETC or their impaired activation led to a substantially delayed wound healing (54, 81, 160–162). Additional roles of DETC include regulation of aberrant inflammation in a model of contact dermatitis (55) and protection against UV-mediated DNA damage (165), cutaneous infection (56) and development of malignancies (57, 58, 166). Interestingly, DETC may mediate their anti-cancer effect by direct cytolytic activity in a TCR- and NKG2D-dependent manner *in vitro* (57). Additionally, IL-13 production by DETC favors the production of IgE (158), that promotes protective anti-cancer immunity through a yet undetermined mechanism involving tumor infiltrating FcεRI^+^ cells (166).

Mucosal and epithelial sites are not only patrolled by natural resident cells like DETC, they are also kept under the surveillance of pathogen-induced CD8^+^ and CD4^+^ αβ T_RM_ cells which provide local long-lived protection against reinfection (7, 8). Natural and induced resident T cells occupy a similar space. Cutaneous infection by herpes simplex virus (HSV) generates CD8^+^ T_RM_ that remain in the basal epidermis around the lesion site (167, 168). Surprisingly, the increased CD8^+^ T_RM_ density at the site of infection inversely correlated with DETC numbers even several months after pathogen clearance. Conversely, distant DETC-rich areas had a reduced CD8^+^ T_RM_ population. One potential explanation for the redistribution of resident T cell subsets is that infection may lead to selective loss of DETC, creating a niche for CD8^+^ T_RM_ cell seeding. Indeed, DETC are rapidly infected by HSV after cutaneous exposure (169). HSV infection of non-neuronal cells is typically lytic and may induce their death. However, alternative mechanisms may also lead to loss of DETC as their redistribution was also observed after intradermal injection of effector CD8^+^ T cells in the absence of infection (168). DETC can also be temporarily displaced by infiltrating NKT cells following acute stress (58), demonstrating that conventional and unconventional αβ T cells can colonize the skin and create a niche at the expense of DETC. It has been proposed that these cells may compete for maintenance signals like IL-15 or aryl hydrocarbon receptor (AhR) ligands (170), which are necessary for mature DETC survival in the skin (171–174). Such competition should also occur between αβ T_RM_ generated by different, non-overlapping infections as both populations would be expected to have similar homeostatic requirements. However, it was recently reported that the generation of new αβ T_RM_ cells does not result in the replacement of previously established T_RM_ cells (175), suggesting that limited resources like IL-15 may not be responsible for redistribution of DETC and αβ T_RM_ cells. Identifying the factors involved in the maintenance of natural and induced T cell populations is necessary to better understand their apparent competition and would be beneficial for the design of targeted local therapies.

### Intestinal intraepithelial lymphocytes–γδ IEL

The intestinal epithelium is actively patrolled by IEL, a large fraction of which are unconventional γδ T cells expressing a CD8αα homodimer in mice (19, 176). The intestine is colonized by γδ IEL during the perinatal period. In contrast to the essential role of the thymus in the generation other γδ T cell subsets, its contribution to intestinal γδ IEL development is more limited. Intestinal γδ IEL can develop extrathymically in athymic mice but at lower numbers than in euthymic animals (177–180). IL-7 production has been shown to be fundamental for γδ IEL thymic and extrathymic intestinal development (181, 182). A large fraction of γδ IEL express the Vγ5 TCR (79, 183). The preferential expression of Vγ5 is controlled at the chromatin level by IL-15-STAT5 signals, which regulate the accessibility of the Vγ5 gene and favor its expression in thymocytes and immature IEL (184). Despite the overrepresentation of the Vγ5 TCR among γδ IEL, the overall γδTCR repertoire in the intestinal epithelium is diverse. Indeed, several mechanisms contribute to the diversity of intestinal γδ IEL including various Vδ and Vγ chain pairings, usage of the Jδ1 or Jδ2 segment and addition of non-germline encoded nucleotides (79, 183). Because of their TCR heterogeneity, γδ IEL have the potential to recognize a wide array of potential antigens or ligands that include host-derived molecules such as nonclassical and nonpolymorphic MHC class Ib molecules T10 and T22 (185). Despite the similarity to MHC class I molecules, T10 and T22 do not present peptide antigens. T10/T22 reactivity is conferred by a specific W-(S)EGYEL CDR3δ motif, which allows some Vγ5^+^, Vγ1.1^+^ and Vγ2^+^ γδ IEL to bind T10/T22 (185). To date, the antigenic specificity of the non-T10/T22 reactive γδ IEL remains obscure.

γδ IEL precursors do not require S1P1 for their emigration from the thymus (186). However, γδ thymocytes and unconventional (CD8αα^+^) recent thymic emigrants express high levels of the gut homing receptors CCR9 (187, 188) and α_4_β_7_ integrin (187–189). Interestingly, CCR9 is preferentially expressed by antigen-inexperienced CD122^lo^ or CD62L^hi^ CD44^int/lo^ thymocytes (189, 190), suggesting they have more potential to home to the gut and that some γδ IEL did not encounter their antigen prior to their migration into intestinal tissues. This assumption was confirmed by the presence of similar numbers of T10/T22 reactive γδ T cells in the intestinal epithelium of *B2m*^−/−^ mice, which lack surface expression of T10/T22 (190). Intestinal γδ IEL might be selected based on their TCR affinity more than their specificity, as suggested by the inverse correlation between TCR affinity and CCR9 expression (190). This unusual “non-selection” of a diversified γδ T cells likely reflects the need to maintain a heterogeneous broadly reactive population that can respond appropriately to the wide variety of stresses and antigens encountered in the intestine.

Within the first few weeks of life, Vγ5^+^ T cells expand in the intestinal epithelium and transition from an immature to a mature phenotype (180). Despite the heavy microbial colonization of the gut, γδ IEL expansion and maturation are independent of the microbiota (66, 178). Instead, expansion and maturation are regulated in a TCR-dependent manner by the BTN-like (Btnl)1 and Btnl6 heterocomplex expressed on the surface of enterocytes (180), reminiscent of Skint-1-mediated selection of DETC in the thymus (144–146). Upon selection by cells co-expressing Btnl1 and Btnl6, Vγ5^+^ T cells upregulate CD25 and produce pro-inflammatory cytokines like IFNγ, growth factors like GM-CSF and chemokines like CCL4 (180). The Btnl-mediated selection of intestinal γδ IEL may occur in a similar fashion in humans, with Vγ4^+^ T cells being activated by cells co-expressing BTNL3 and 8 (180). Once established in the tissue, γδ IEL rely on the production of IL-15 by microbiota stimulated intestinal epithelial cell (IEC) (191–193) and AhR ligands (174) for their maintenance and survival. In return, γδ T cells participate in the maintenance of tissue homeostasis and barrier integrity. γδ IEL promote IEC proliferation and maturation through multiple mechanisms that may include production of KGF (83, 129, 141), regulating tight junctions (67), producing anti-microbial peptides in response to pathobiont invasion (68), limiting tissue damage, and promoting epithelial repair after injury (141).

γδ IEL from specific pathogen-free (SPF) mice constitutively express cytotoxic genes, including granzyme A and B (194), and can lyse target cells directly *ex vivo* (195), consistent with an anti-infectious role of intestinal γδ IEL. The absence of γδ T cells in *Tcrd*^−/−^ was associated with enhanced dissemination of enteric bacteria (*Salmonella enterica* serovar Typhimurium) or parasites (*Toxoplasma gondii*), rendering mice more susceptible to systemic infection (67–69). Additionally, γδ IEL indirectly protect from murine norovirus infection by secreting type I and III interferons and increasing the resistance of IEC to viral infection (196). They are also important in controlling dissemination of commensals that may occur with loss of barrier integrity after pathogen invasion or epithelial injury (197). Thus, γδ IEL serve multiple functions in regulating immunity at the mucosal interface with the environment.

Intestinal γδ IEL were initially thought to have limited mobility within the epithelium (188). This view has recently been challenged by two compelling studies that demonstrated that intestinal γδ IEL are highly dynamic and constantly migrate within the intestinal tissue. During tissue homeostasis, individual γδ IEL survey a large surface area and contact numerous IEC within a short period of time (66, 198). γδ IEL mainly remain in the middle region of the intestinal villi, between the basement membrane and the epithelial layer, but they also appear to occasionally migrate to the intercellular space between IEC for a short period of time (66, 198). Although commensals do not impact γδ IEL numbers, microbial colonization is required for their normal distribution within the villi and their migratory behavior in the tissue (66), and also promotes their cytotoxic and anti-microbial functions (68, 195). These patterns drastically change upon enteric infection with invasive bacteria or parasites. Shortly after pathogenic exposure, γδ IEL preferentially localized to pathogen-rich areas and decreased their normal surveillance behavior. Reduced surveillance coverage was associated with increased movement between IEC and the lateral intercellular space in a behavior termed “flossing” (66, 69) that is regulated by the tight junction protein occludin (198). These behavioral and functional changes result from the MyD88-dependent sensing of pathogenic microbes by IEC, and the specific abrogation of MyD88 signaling in IEC severely blunted γδ IEL responses (66, 68). γδ IEL at steady-state may also be activated through their TCR as injection of a TCRδ-specific antibody diminished intracellular calcium flux (199). It is therefore conceivable that the IEC-γδ IEL dialogue could also involve TCR-mediated tissue surveillance. Thus, γδ IEL continually survey epithelial integrity via cross-talk with IEC which dictates γδ IEL behavior and leads to their adaptation in the intestinal environment. While the exact function of γδ IEL flossing remains unclear, its association with pathogen hotspots and the importance of γδ T cell responses to anti-infection immunity suggests an important role of flossing in controlling intestinal infections or promoting epithelial repair.

Natural tissue-resident γδ T cells are remarkably adapted to their tissue of residence, where they provide signals necessary to maintain tissue homeostasis and barrier integrity while also providing a rapid front-line defense against infectious assaults continually encountered in epithelial tissues. Both DETC and intestinal γδ IEL are adapted to efficiently survey their respective tissues, through their placement/migration into the tissue and communication with neighboring epithelial and immune cells. Despite this, natural tissue-resident T cells may have to compete for limited space or nutrients with *de novo* generated conventional T_RM_ cells after local infections. Whether direct competition for resources and space or an undefined crosstalk between these cells regulate tissue colonization is unclear and an area of much interest.

## Microbiota-induced γδ17 T cells: diversified effectors with multifaceted roles

Almost all tissues exposed to the environment are colonized by established commensal communities, with the exception of the eye for which the presence of a resident microbiome remains a matter of debate (1). The presence of these microorganisms shapes the local immune system and promotes protective anti-infectious immunity, as exemplified by the anti-bacterial, -fungal or -parasitic type-17 and type-1 responses triggered by segmented filamentous bacteria in the intestines (2) or *Staphylococcus epidermidis* (*S. epidermidis*) and other commensals in the skin (3, 4), respectively. However, commensal-specific T cells (especially intestinal T_H_17 cells) can also have detrimental effects at remote sites under certain circumstances, inducing pathological inflammatory responses that lead to the development of diseases like arthritis and autoimmune encephalomyelitis (5, 6).

As for conventional T cells, the microbiota also impacts γδ T cell responses at many body sites. Interestingly, commensal-induced γδ T cell responses appear to largely involve IL-17A-producing cells regardless of their tissue distribution among diverse sites such as the skin (4, 200), the liver (22), the oral and peritoneal cavities (23, 201), the eye (24), the lungs (28) and the intestines (29, 197). The generation and activation requirements of microbiota-induced γδ T cells appear uniquely adapted to the tissue location. First of all, the presence of a microbiota is a prerequisite for the development of some, but not all, tissue tropic γδ T cells. Indeed, antibiotic-treated SPF or germ-free (GF) mice harbor fewer activated liver-resident (22), pulmonary (28), peritoneal, and small intestinal lamina propria (siLP) γδ17 T cells (23). In contrast, γδ IEL numbers are independent of a microbiota (66, 178, 197). Second, few identified microorganisms have been specifically associated to particular γδ T cell populations: *Corynebacterium mastidis* (*C. mastidis*) colonization with ocular Vγ2^+^ γδ17 T cells (24), *Corynebacterium accolens* (*C. accolens*) and other bacteria from the *Corynebacterium* genus producing mycolic acid with skin Vγ2^+^ γδ17 T cells, and *S. epidermidis* with skin Vγ2^−^ γδ17 T cells (200). The expansion of Vγ2^+^ and Vγ2^−^ γδ T cell subsets by *C. accolens* and *S. epidermidis* association, respectively, demonstrates that the γδ T cell responses can adapt within the same niche. In contrast, other γδ T cell subsets only require the presence of a microbiota without any distinction between bacterial species (22, 28). Lastly, many different signals control the activation and/or expansion of commensal-induced γδ17 T cells, including lipid presentation by the non-classical molecule CD1d (22), DC-mediated expansion (24, 201) and activation/polarization (27, 29, 200) or MyD88 signaling pathways (23, 197). Cytokines like IL-1β (23, 24), IL-23 (200) and IL-6 (28), either alone or in combination with other activation signals, also participate in the induction or propagation of IL-17A from microbiota-induced γδ T cells.

IL-17 family cytokines, including IL-17A, are key regulators of mucosal and epithelial immunity. Over the past decade, a multitude of roles, from the induction of protective anti-infectious responses to the promotion of pathological inflammatory processes, have been attributed to IL-17A (202). Accordingly, the induction of γδ17 T cells by microbial colonization has also been associated with seemingly contrasting effects. Commensal-induced γδ T cells can mediate local protection against penetrating commensals (197), pathogenic bacteria or even yeast, as exemplified by the resistance displayed by *C. mastidis* colonized animals to ocular *Candida albicans* infection (24). In this model, induced γδ T cells were driving the production of antimicrobial peptides such as S100A8 and S100A9 and the recruitment of neutrophils through the production of IL-17A. As IL-17A can elicit these responses in virtually all mucosal and epithelial surfaces, similar broad-spectrum anti-infectious immunity might occur in other γδ T cell rich tissues. In contrast to their protective effect against infection, microbiota-elicited γδ17 T cells may be beneficial (28) or harmful (29) in cancer. Other local detrimental effects attributed to microbiota-induced γδ17 T cells include the acceleration of nonalcoholic fatty liver disease by liver-resident γδ17 T cells (22) and the exacerbation of imiquimod-induced skin inflammation following *C. accolens* association (200).

Microbiota-elicited γδ T cells can also impact distal immune function. They express a plethora of homing receptors that allows them to navigate to distant tissues and impact health or disease. For example, γδ T cells are recruited to the ischemic penumbra after ischemic stroke in a CCR6-dependent manner (203). There, they contribute to exacerbate brain injury through the production of IL-17A and subsequent recruitment of neutrophils (203–205). In a recent study using a transient middle cerebral artery occlusion mouse model, the γδ17 T cells recruited to the ischemic brain originated from the small intestine and were dependent on specific commensal species for their maintenance (27). Alteration of the gut microbiota by antibiotic treatment led to a reduction in intestinal γδ17 T cells and diminished γδ T cell infiltration to the meninges, limiting injury. Thus, commensal-induced γδ T cells may have local and distal effects on pathological or physiological tissue processes.

It is now well established that the microbiota is a critical component of human health and disease. In addition to providing many enzymatic and metabolic pathways and colonization resistance to invading pathogens, commensals also participate in the development of and shaping of the immune system (206). Dysbiosis can be sensed by the immune system and has been associated with the development or exacerbation of many diseases in many organ systems. Given their preferential association with epithelial and mucosal tissues, it is not surprising that some γδ T cell populations are also influenced by the microbiota.

## Inflammatory disease and memory-like γδ17 T cell response

**Figure 1:**
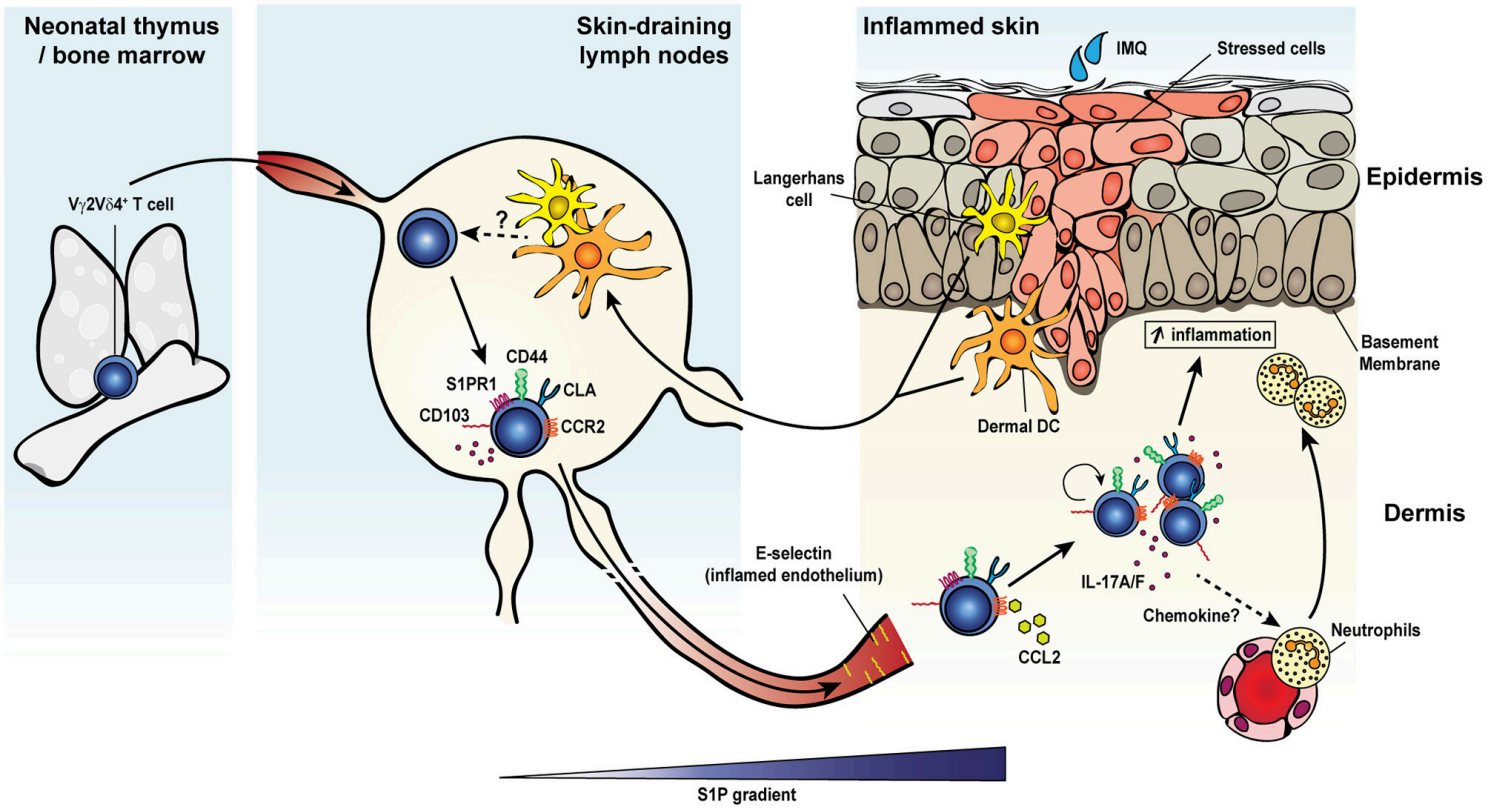
Inflammation-induced dermal memory γδ T cells sensitize mice to imiquimod-induced psoriasis. Topical skin exposure of naïve mice to the TLR7/8 ligand imiquimod (IMQ) activates regional dendritic cells (Langerhans cells or dermal DCs) which migrate to the draining lymph nodes and present antigens to Vγ2Vδ4^+^ T cells. Activated γδ T cells proliferate, acquire an effector/memory CD44^hi^ CD27^−^ CD62L^−^ phenotype and upregulate several migration molecules favoring their egress from the lymph nodes (S1P1) and homing to the inflamed and resting skin dermis (CCR2 and cutaneous lymphocyte-associated antigen or CLA), where the cells establish memory. Secondary IMQ skin application at the same or a distant site leads to the local proliferation and activation of dermal memory Vγ2Vδ4^+^ T cells, which produce large amount of IL-17A/F and promote the recruitment of neutrophils and thereby exacerbate skin inflammation.

In addition to γδ T cell responses to the microbiota or after infection, γδ T cells have also been implicated in innate responses in inflammatory disease. Inflammatory diseases with γδ T cell contributions include multiple sclerosis or EAE (72), psoriasis (135), collagen induced arthritis (73), ankylosing spondylitis (74), inflammatory bowel disease (63, 64), and uveitis (75). One factor of inflammatory disease progression attributed to γδ T cells is IL-17A production, a feature often associated with changes in the microbiota (72, 73, 135). Inflammation-induced tissue damage may allow bacteria to bypass the epithelium leading to a positive feedback inflammatory loop. Interestingly, memory-like γδ T cell formation has been seen in inflammation of the skin (25, 26, 51, 207). IL-17A-producing Vγ2Vδ4^+^ T cells initially derive from the neonatal thymus where they are instructed with tissue tropism. IMQ-induced psoriasis-like skin inflammation triggers a potent long-lived Vγ2Vδ4^+^ T cell response (Figure 1) (25, 26). These Vγ2Vδ4^+^ T cells were phenotypically memory-like with a CD44^hi^ CD62L^lo^ CD27^−^ expression pattern. Vγ2Vδ4^+^ T cells expanded after primary challenge and migrated from the draining lymph nodes to both the inflamed and uninflamed skin in a S1P1-dependent manner where they persisted. Migration of Vγ2Vδ4^+^ T cells from the circulation to the skin may also be influenced by signals including cutaneous lymphocyte antigen (CLA) binding to P- and E-selectins, CD103 interactions with E-cadherin, and C-C chemokine receptor type 2 (CCR2), and CCR6. CCR2 appeared essential for γδ17 T cell recruitment to inflamed tissues in B16 melanomas and EAE while CCR6 appeared necessary for dermal γδ17 T cell residence (208). Subsequent IMQ administration on previously untreated skin induced an accelerated and robust re-expansion of skin resident Vγ2Vδ4^+^ T cells that produced IL-17A/F and exacerbated disease (25, 26). IL-17 production and subsequent neutrophil recruitment for skin disease appeared be partially dependent on an NFκB-inducing kinase (207). Enhanced inflammation with subsequent exposure was also associated with the Vγ2Vδ4^+^ T cell recall response but independent of αβ T cells (26). These findings were also noted in an acute contact dermatitis model using dinitrofluorobenzene where a similar memory Vγ2^+^ γδ17 T cell population appeared predominately tissue-resident in classical parabiosis experiments (51). Together, these studies suggest that γδ T cells can modulate inflammatory diseases of the skin by forming long-lived tissue resident memory populations that exacerbate disease through the production of IL-17 family cytokines. While these studies suggest the establishment of long-lived memory T cells, whether this response is driven by a specific antigenic responsiveness or is broadly reactive is unclear.

## Infection-induced adaptive γδ T cells: long-term players in mucosal immunity

Anamnestic immunity was thought to be mediated solely by conventional αβ T cells and B cells. The recent identification of several innate and unconventional memory responses challenged this belief and has reshaped our view of immunological memory. γδ T cells bridge innate and adaptive immunity in many contexts by rapidly responding to stresses such as infections and promoting conventional adaptive immunity. For that reason, most mouse studies focused on γδ T cell responses in the first few hours to days after pathogen exposure or inflammatory insult. However, mounting evidence in humans, non-human primates and mice demonstrated that γδ T cells can mount adaptive-like responses. One of the most studied pathogens in that context is CMV. Indeed, the involvement of γδ T cells in the protective response to CMV infection was first suggested in kidney transplant patients whose γδ T cells underwent a massive and long-lasting expansion in the blood (34, 209, 210). γδ T cell expansion to CMV was also observed in the context of immunosuppression or immunodeficiency (35, 36, 126, 211–215), neonatal infection (216) and in otherwise healthy individuals (35, 125). Analysis of the repertoire of CMV-selected γδ T cells revealed an oligoclonal and in some individuals even monoclonal population (34, 35, 125), which, surprisingly, did not involve circulating Vγ9Vδ2^+^ T cells but tissue tropic Vδ2^−^ γδ T cells. Expanded cells displayed a T_EMRA_ phenotype, similar to CMV-specific CD8^+^ T cells (127), and only responded to CMV infection (34, 128). Importantly, the expansion of Vδ2^−^ γδ T cells correlated with the resolution of the acute infection in humans (210) and adoptive transfer of murine CMV-expanded γδ T cells conferred full protection to susceptible immunodeficient mice (217, 218). Thus, CMV-elicited γδ T cells display many features classically attributed to conventional memory T cells. Another long-lived γδ T cell response to virus has been reported in the context of vaccinia virus immunization in humans (38) and rhesus macaques (39). Interestingly, vaccinia virus immunized macaques were protected against monkeypox virus challenge infection and this was associated with the expansion of circulating and pulmonary Vγ9Vδ2^+^ T cells. Long-lasting adaptive-like γδ T responses were also reported in the circulation of individuals infected with the protozoan *Plasmodium falciparum* (*P. falciparum*) (40–43) and the circulation and peripheral tissues of animals infected with *Plasmodium chabaudi* (44). Interestingly, γδ T cell distribution to parasite-targeted tissues raises the possibility that these cells might provide unique functions to control parasite replication during the blood and liver stages. Collectively, these studies provide compelling evidence of adaptive γδ T cell responses triggered by unrelated pathogens in humans, non-human primates and rodents. However, the chronic or latent nature of the infections and their associated antigen and inflammation in conjunction with some inherent challenges associated with human studies has hindered conclusive demonstrations of the memory potential and long-term tissue residency of these populations.

### Infection-induced *bona fide* memory γδ T cell responses

Adaptive γδ T cells survey exposed mucosal and epithelial barriers where they may participate in pathogen clearance or control and have tissue-adapted functions. γδ T cells are one of the first immune responders in many bacterial infections, where they act concurrently with cells of the innate immune system. However, this innate γδ T cell response does not preclude the establishment of a subsequent localized memory γδ T cell response. A mouse model of peritonitis induced by repeated intraperitoneal exposure to *Staphylococcus aureus* (*S. aureus*), induced a rapid Vγ1.1^+^ and Vγ2^+^ γδ17 T cell response in the peritoneum and the draining mediastinal lymph nodes a few hours after exposure (49). After this early polyclonal innate response, a long-lived predominantly IL-17A-producing Vγ4^+^ T cell population emerged in both tissues. Surprisingly, secondary challenge with *S. aureus* of previously exposed but pathogen-free mice induced a conventional memory response of Vγ4^+^ T cells. Recalled Vγ4^+^ T cells underwent secondary expansion, displayed an activated CD44^hi^ CD27^−^ phenotype, and produced elevated levels of IL-17A. Adoptive transfer of purified *S. aureus*-elicited Vγ4^+^ T cells was sufficient to protect naïve recipients against peritonitis and bacterial dissemination to the liver and kidneys (49). In contrast to the fundamental role of IL-1β in the induction of IL-17A production by naive γδ T cells during primary *S. aureus* exposure, memory Vγ4^+^ T cells were IL-1β-independent suggesting that memory γδ T cells have an altered ability to respond to unique environmental cues to provide effector functions. Localized *S. aureus* infection of the skin in *Il1b*^−/−^ mice resulted in poor bacterial control during primary infection but protection against reinfection, revealing the potential presence of an additional memory γδ T cell subset. Indeed, intradermal infection induced the selective expansion of skin resident Vγ4Vδ1^+^ and Vγ3Vδ1^+^ T cell clones with conserved CDR3δ and CDR3γ motifs that were maintained during the convalescent phase and present after secondary infection of WT and *Il1b*^−/−^ mice (50). Protection during secondary infection was conferred by IFNγ- and TNFα-producing γδ T cells. Adoptive transfer of purified *S. aureus*-elicited γδ T cells, but not CD4^+^ T cells, neutrophils or serum from convalescent mice, was associated with bacterial clearance. Thus, different memory γδ T cell responses can be induced by the same pathogen and local memory γδ T cell populations may be tissue adapted to provide distinct protective mechanisms.

**Figure 2:**
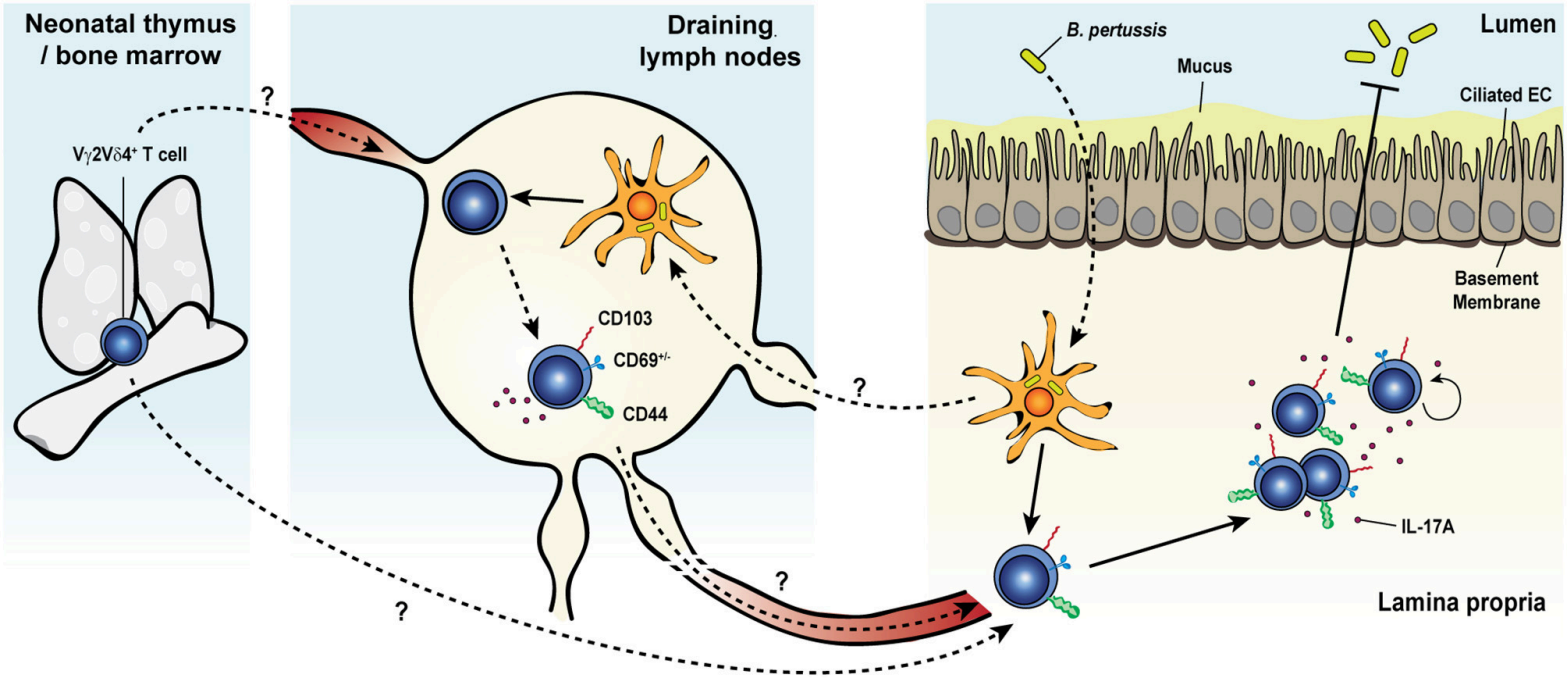
Memory γδ T cell response to pulmonary *Bordetella pertussis* infection. Upon primary intranasal infection with *Bordetella pertussis* (*B. pertussis*), Vγ2Vδ4^+^ T cells are activated by *B. pertussis* antigen-presenting dendritic cells either in the draining lymph nodes or directly in the lung tissue. Activated γδ T cells expand, display a CD44^+^ and CD103^+^CD69^+/−^ activated resident memory phenotype and remain at an elevated number in the lungs after bacterial clearance. Secondary exposure to *B. pertussis* induces a recall expansion of memory Vγ2Vδ4^+^ T cells in the lung tissue and a protective and robust IL-17A response leading to an enhanced pathogen clearance.

In addition to the memory responses involving Vγ4^+^ T cells, a long-lasting protective response of Vγ2^+^ T cells was observed after pulmonary *Bordetella pertussis* (*B. pertussis*) infection (Figure 2) (48). After an early innate response dominated by IL-17A-producing Vγ1.1^−^ Vγ2^−^ γδ T cells, effector memory CD44^+^ CD27^−^ Vγ2^+^ T cells started accumulating from day 14 and were maintained long-term in the lungs. The later emergence of Vγ2^+^ T cells coincided with the expansion of T_RM_ precursors and T_EM_-like CD4^+^ T cells in the lungs (219). Expanded pulmonary Vγ2^+^ T cells share several features with *B. pertussis*-specific memory CD4 T cells: (i) they reside in the lungs for a prolonged period of time after bacterial clearance and rapidly and locally proliferated in response to secondary pulmonary challenge, (ii) a considerable fraction expresses the T_RM_ marker CD69 and some also co-express CD103, (iii) they have a strict reactivity to *B. pertussis*, (iv) they are biased toward IL-17A production, and (v) they contribute to enhanced bacterial clearance after challenge (48, 219). Thus, *B. pertussis*-elicited memory γδ T cells closely resemble conventional T_RM_ cells. In contrast to the reported displacement of skin DETC by virus-specific CD8^+^ T_RM_ (168), CD4^+^ T_RM_ and memory γδ T cells were able to coexist in the lungs of infected mice and both subsets expanded after infection and participated in conferring protection, suggesting that they may reside in distinct niches within the tissue or do not compete for space or survival factors.

Microorganisms producing PAgs are potent activators of human and non-human primate Vγ9Vδ2^+^ T cells. Mycobacteria, including *Mycobacterium bovis* BCG strain and *Mycobacterium tuberculosis* (*M. tuberculosis*), produce HMBPP (220–222), the most potent Vγ9Vδ2^+^ T cell activator. Correspondingly, intravenous (i.v.) BCG vaccination of macaques triggered a drastic expansion of these circulating cells in the blood, but also in the lungs and the intestines (45). Pulmonary *M. tuberculosis* infection led to a similar expansion of mucosal but not circulating Vγ9Vδ2^+^ T cells (47), demonstrating tissue-adapted responses by adaptive γδ T cells that may be predicated on immunization route. BCG challenge of vaccinated monkeys induced a more rapid and robust clonal expansion of Vγ9Vδ2^+^ T cells but no other γδ T cell subsets. Thus, Vγ9Vδ2^+^ T cells are capable of forming long-lived clonally-expanded memory responses (45). Interestingly, direct contact with antigen presenting cells was required for the recall-like expansion of Vγ9Vδ2^+^ T cells (46). The recall response of Vγ9Vδ2^+^ T cells in BCG immunized macaques was associated with enhanced clearance of challenge infection and protection against fatal tuberculosis (45). In line with these findings, Vγ9Vδ2^+^ T cells induced in BCG vaccinated volunteers that were previously unexposed to any *mycobacteria* showed an enhanced responsiveness to *M. tuberculosis ex vivo* (223), suggesting that BCG vaccination also primes γδ T cells to respond to *M. tuberculosis* in humans. Although human and monkey Vγ9Vδ2^+^ T cells share many features, including a memory-like response to *mycobacteria*, it remains to be established whether human γδ T cells, like their non-human primate counterparts, are maintained in peripheral tissues following BCG immunization to confer some protection against *M. tuberculosis* infection.

### Multifunctional memory γδ T cells to *L. monocytogenes*

*L. monocytogenes* is known to be a potent inducer of γδ T cell responses. In humans, expansion of Vγ9Vδ2^+^ T cells has been detected in the blood of pregnant women, newborns, infants and the elderly early after *L. monocytogenes* exposure (224, 225). These γδ T cells displayed an activated (HLA-DR^+^) and memory (CD45RO^+^) phenotype. Consistent with a predetermined innate response, stimulation of PBMC from healthy donors with heat-killed *L. monocytogenes* (225), listeria lysate or culture supernatant (226) led to rapid proliferation of Vγ9Vδ2^+^ T cells.

A similar mobilization of circulating γδ T cells during *L. monocytogenes* infection has also been reported in rhesus macaques. In a model of disseminated *L. monocytogenes* infection, Vγ9Vδ2^+^ T cells increased in the blood of rhesus macaques infected with an attenuated *L. monocytogenes* strain through an intramuscular, and to a lesser extent i.v. route (59). These cells were also elevated in bronchoalveolar lavages and rectal biopsies suggesting that they actively traffic to and seed mucosal tissues during infection. More interestingly, *L. monocytogenes* challenge of immunized animals led to a rapid and robust re-expansion of Vγ9Vδ2^+^ T cells that correlated with the resolution of infection (59). One peculiar feature of *L. monocytogenes* is its ability to use both the classical mevalonate and the alternative MEP pathways for isoprenoid synthesis (227). Both primary and recall-like responses of Vγ9Vδ2^+^ T cells have been shown to rely on the bacteria's ability to co-produce mevalonate-derived isopentenyl pyrophosphate and MEP-derived HMBPP, the latter being much more efficient at inducing primary and secondary expansion of primate Vγ9Vδ2^+^ T cells and promoting their differentiation into CD27^+^ CD45RA^−^ CD28^−^ memory cells (60). *L. monocytogenes*-elicited γδ T cells displayed various effector functions after secondary challenge, including production of IFNγ, IL-4, IL-17A, and TNFα (59). Surprisingly, a substantial portion of these cells were multifunctional and simultaneously produced IFNγ and IL-17A, IFNγ and IL-4, or TNFα and perforin in response to HMBPP (59, 60). Expanded Vγ9Vδ2^+^ T cells were also potent bactericidal effectors capable of efficiently lysing *L. monocytogenes*-infected DC and restraining intracellular bacterial growth in macrophages *ex vivo*. Thus, *L. monocytogenes* infection elicits a multifunctional circulating γδ T cell response in non-human primates. Because this response is accompanied by the colonization of epithelial tissues, infection-elicited mucosal γδ T cells may also have distinct effector functions that provide tissue-adapted responses.

**Figure 3:**
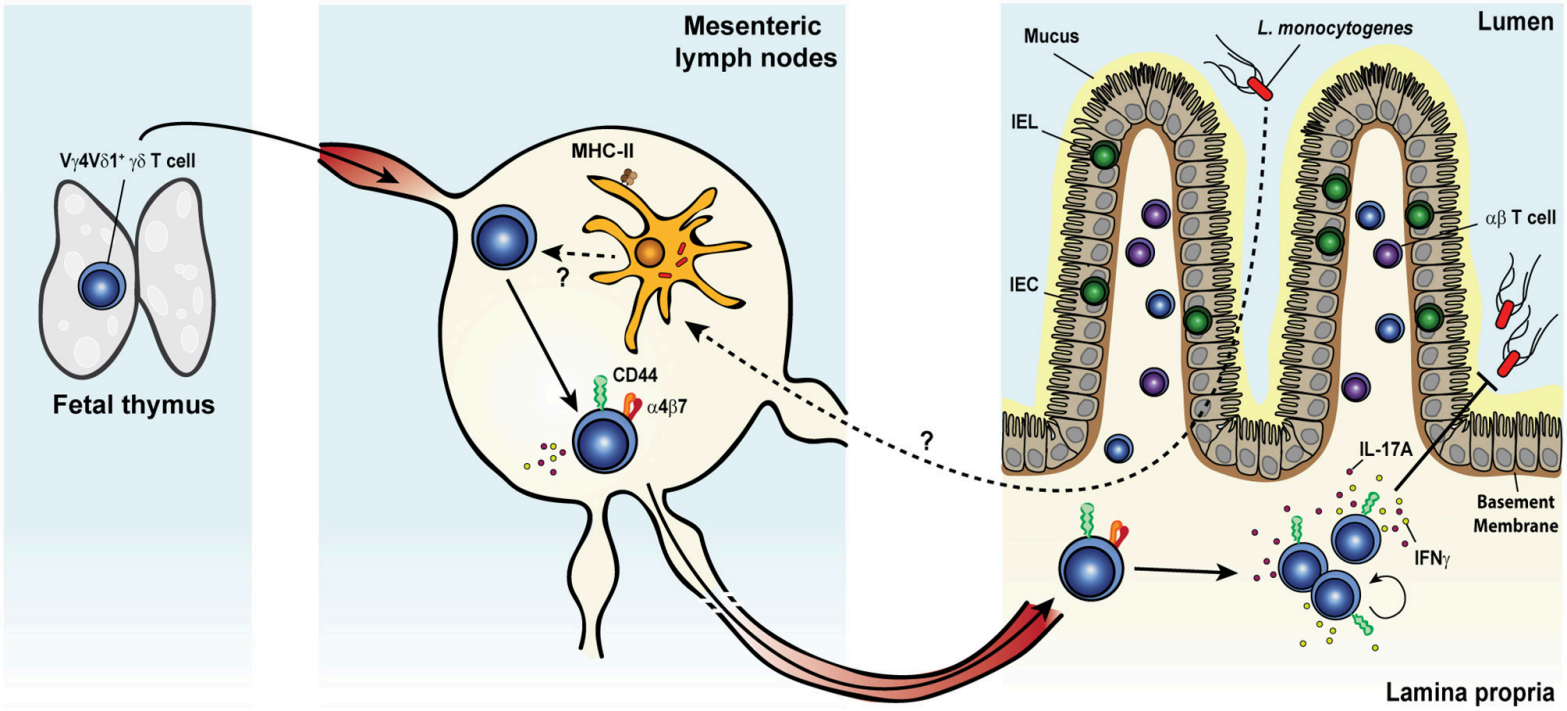
*Listeria monocytogenes* elicits a multifunctional protective memory response from fetal γδ T cells. Shortly after oral infection, the foodborne pathogen *Listeria monocytogenes* (*L. monocytogenes*) crosses the intestinal barrier and migrates to the mesenteric lymph nodes (MLNs). Whether *L. monocytogenes* reaches the MLNs extracellularly or is carried intracellularly by migratory intestinal dendritic cells is yet unclear. Colonization of the MLNs leads to the expansion of a population of semi-invariant Vγ4Vδ1^+^ T cells characterized by a CD44^hi^ CD27^−^ phenotype and the rare ability to co-produce both IL-17A and IFNγ, in a process that would likely involve MHC-II^+^ cells. Activated cells upregulate the gut-homing integrin α_4_β_7_ and migrate through the blood circulation to the intestinal lamina propria (LP). After pathogen clearance, *L. monocytogenes*-elicited γδ T cells become resident memory cells and persist long term in both tissues. Memory γδ T cells undergo a rapid and dramatic re-expansion upon re-exposure to *L. monocytogenes* and cooperate with conventional T cells to confer heightened protection against the bacterium.

A large body of evidence has convincingly demonstrated the involvement of γδ T cells in the early phase of the primary immune response to systemic *L. monocytogenes* infection of mice (228–244) and rats (245, 246). More recently, our group reported a *bona fide* memory γδ T cell response in mice after food-borne infection with a mouse-adapted *L. monocytogenes* capable of intestinal epithelial cell invasion (Figure 3) (62, 134, 247). Food-borne infection induced a long-lived Vγ4Vδ1^+^ T cell population in the gut draining mesenteric lymph node (MLN) with a CD44^hi^ CD27^−^ phenotype (62). By 7 days after infection, these cells were mobilized into the blood, up-regulated the gut-homing integrin α_4_β_7_ and trafficked to the intestinal lamina propria similarly to conventional *L. monocytogenes*-specific CD8^+^ (248) and CD4^+^ (249) αβ T cells. Like *L. monocytogenes*-induced CD4^+^ and CD8^+^ αβ T_RM_ cells, *L. monocytogenes*-elicited γδ T cells established residency in MLN and intestinal lamina propria where they were maintained long term in the absence of further antigenic stimulation (62, 134). The generation of this γδ T cell subset was restricted to tissues associated with the gastrointestinal system and was induced by food-borne (62) but not i.v. infection (232, 233). *L. monocytogenes*-elicited γδ T cells demonstrated enhanced anamnestic response upon *L. monocytogenes* challenge infection and were fully competent for immunologic boosting upon tertiary exposure (62). Although *L. monocytogenes*-elicited γδ T cells appeared to share a similar anatomical niche as *L. monocytogenes*-specific CD4^+^ and CD8^+^ αβ T cells (248, 249), all populations expanded robustly after infection and were maintained without any apparent competition for limiting resources or anatomic space.

Memory γδ T cells cooperated with αβ T cells to confer optimal protection in the MLN and the small intestine during food-borne *L. monocytogenes* challenge infection. Indeed, only the concomitant antibody-mediated depletion of αβ T cells (both CD8^+^ and CD4^+^) and forced internalization of the γδ TCR resulted in the complete loss of protection afforded to immunized mice, whereas the sole removal of αβ T cells only partially impaired *L. monocytogenes* control (62). One striking feature of *L. monocytogenes*-elicited γδ T cells was their ability to produce IFNγ and IL-17A during each stage of the immune response. Moreover, subsets within the CD44^hi^ CD27^−^ γδ T cell population co-produced both cytokines during the primary and secondary responses (62), reminiscent of the multifunctional response described in rhesus macaques after secondary challenge (59). During the recall response, the majority of IL-17A was derived from reactivated memory γδ T cells in the MLN. This production of IL-17A was a critical component of anti-listerial immunity as it mediated the formation of *L. monocytogenes*-containing immune cell clusters composed of memory γδ T cells and IL-17RA^+^ inflammatory monocytes and neutrophils (134).

Collectively, these studies demonstrate that systemic and food-borne *L. monocytogenes* infection generates long-lived multifunctional memory γδ T cells in rhesus macaques and mice, respectively. Thus, a population of pathogen-elicited γδ T cells appears to behave very similarly between mice and primates, and this may suggest a conserved biology among mucosal γδ T cells. These studies also highlight the important influence of infection route and models that mimic natural infection on understanding the γδ T cell response. Interestingly, amongst the memory and memory-like responses described to date, *L. monocytogenes* is the only agent known to induce multifunctional γδ T cells in two distinct species. Although γδ17 T cells are known to have a permissive chromatin state for IFNγ expression (102), other memory γδ T cell populations reported in mice only produce IL-17A (25, 26, 48, 49). Conversely, only IFNγ was shown to be produced by virus-activated memory-like Vγ9Vδ2^+^ T cells (39). miR-146a has recently been shown to negatively regulate IFNγ production by murine γδ17 T cells, including during oral *L. monocytogenes* infection (61). Elucidating the mechanisms by which *L. monocytogenes* partially breaks miR-146a-mediated inhibition of IFNγ production by γδ17 T cells and understanding why other pathogens do not would provide important clues about the fine regulation of γδ17 T cell functions and might open new avenues for the manipulation of these cells.

## Anti-tumor memory γδ T cells in cancer

A substantial body of research has focused on the beneficial nature of γδ T cells in anti-cancer immunity and their potential as a targetable therapeutic since a landmark study demonstrated that γδ T cells in the epithelial compartment play a substantial role in prevention of cutaneous carcinogenesis (57). Indeed, the presence of an intra-tumoral γδ T cell gene signature was associated with the single most favorable prognostic indicator of patient outcome for a wide range of cancers (250). γδ T cells can have a wide range of effects ranging from reshaping the tumor microenvironment (251, 252), being integral in promoting a diverse cancer protective IgE repertoire through NKG2D stress surveillance (166), or IFNγ production (52). Substantial effort has focused on resolving the anti-tumor activity of Vγ9Vδ2^+^ T cells, the predominant γδ T cell population in human PBMC, in multiple cancers (253–257). Tissue resident Vδ2^−^ γδ T cells may also substantially contribute to anti-tumor immunity. Vδ2^−^ γδ T cells typically predominate over Vδ2^+^ T cells within tumors (52, 65) as well as in tissues from healthy individuals (120). This Vδ2^−^ γδ T cell population is principally composed of Vδ1^+^ T cells but also contain a significant population of Vδ3^+^ T cells. Due to Vδ2^−^ γδ T cell prevalence in tumor microenvironment, it is likely that this subset also substantially contributes to anti-tumor activity.

Vγ9Vδ2^+^ T cells were previously delineated based on expression of CD45RA and CD27 as naive (CD45RA^+^ CD27^+^) cells or effector and memory T_CM_ (CD45RA^−^ CD27^+^), T_EM_ (CD45RA^−^ CD27^−^), and T_EMRA_ (CD45RA^+^ CD27^−^) cells (117). While naive T cells and T_CM_ cells primarily reside in secondary lymphoid tissues, T_EM_ and T_EMRA_ migrate to inflammatory sites to perform effector functions. These latter populations have been investigated in multiple cancers including squamous cell carcinoma (SCC) (52), CRC (65), neuroblastoma (71), and melanoma (53) due to their proliferative capacity and tendency to migrate toward inflammatory sites. Substantial effort has also sought to leverage the anti-tumor properties of Vγ9Vδ2^+^ T cells using approaches like *in vitro* expansion of patient-derived γδ T cells and chimeric antigen receptor T cells for potential adoptive immunotherapies (258, 259). Vγ9Vδ2^+^ T cells can be selectively activated through PAgs or amino bisphosphonates such as zoledronic acid (zoledronate) in combination with various growth factors, cytokines, or costimulatory molecules (260). While various adoptive transfer methods have been primarily explored in a number of pre-clinical studies (261–267), to date, clinically favorable outcomes appear limited to prostate cancer (137). However, challenges remain in the rapid and robust generation of the large numbers of cells that would be necessary for successful adoptive immunotherapies (268). Zoledronate also has various indirect effects on γδ T cells by independently impacting the tumor microenvironment (251, 269, 270), which can provide a pro-tumor or anti-tumor outcome (271, 272). As such, it will be important to assess the contribution of γδ T cells and the impact of any therapies in individual tumor types.

**Figure 4:**
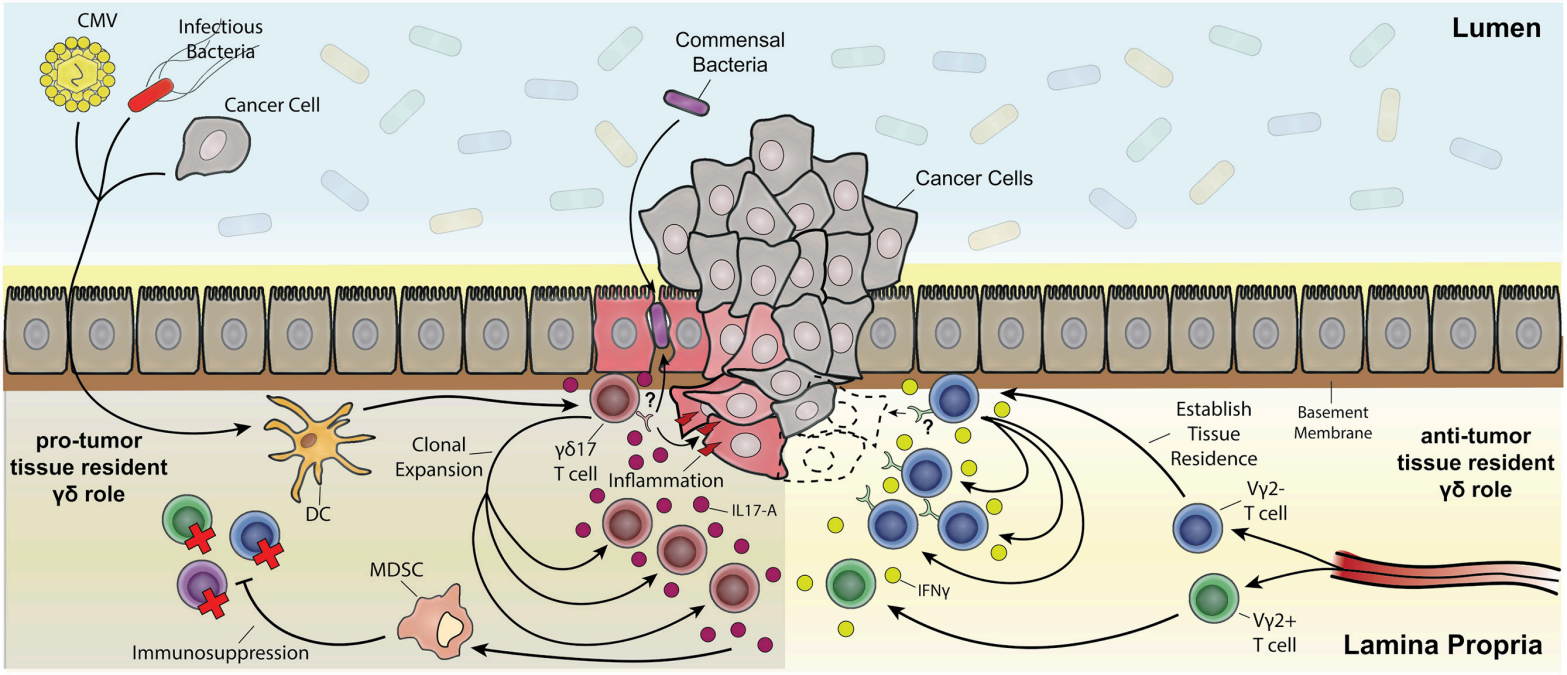
The multifaceted role of resident memory-like γδ T cells in tumorigenesis. Depicted are Vδ2^−^ γδ T cells establishing tissue residency upon being primed by various means (e.g., CMV, bacterial infection, and tumor associated antigens) through localization from the draining lymph nodes to the tissue's epithelial layer. Vδ2^+^ T cells also localize to the tissue but do not establish permanent residence. Both Vδ2^+^ and Vδ2^−^ γδ T cell subsets can be polarized from IFNγ anti-tumor subsets toward pro-tumor IL-17A-producing subsets through inflammatory dendritic cell cytokine signaling (e.g., IL-23). One possibility is that pro-inflammatory tissue damage causes a leaky barrier to commensals and other bacteria and a positive feedback loop of inflammation resulting in expansion of IL-17A-producing γδ T cell subsets. Chronic inflammatory exacerbation opens the window for cancer upon mutagenesis due to constant tissue regeneration. IL-17A signaling also causes myeloid-derived suppressor cells (MDSC) to have an immunosuppressive effect on effector T cells. On the other hand, IFNγ-producing tissue resident Vδ2^−^ subsets clonally expand upon recognition of antigen (in part through stress recognition but it has yet to be thoroughly elucidated) causing tumor cell death.

A protective role of tissue resident γδ17 T cells has been readily described in the context of infectious disease, but they have also been implicated in exacerbating chronic inflammatory diseases like psoriasis. Chronic inflammatory disease is a risk factor and clinical precursor to a number of cancers including pancreatic cancer (273), skin cancer (274) and CRC (275). A growing body of literature has also demonstrated a γδ T cell response that promotes tumor growth. This pro-tumor outcome of some γδ T cell responses appears predominately a consequence of IL-17A production that is often associated with the up-regulation of proliferation pathways in cancerous lesions (276) (Figure 4). These apparent anti- and pro-tumor discrepancies are likely due to the dichotomous functional outcomes associated with type-1 or type-17 γδ T cell responses. A pro-tumor role of IL-17A-producing γδ T cells is evident in a number of cancers such as SCC (52), CRC (29), and metastatic breast cancer (70). In human SCC, tumor infiltration of IL-17A-producing Vδ1^+^ and Vδ2^+^ T cells was associated with a negative prognosis, in contrast to a more favorable outcome associated with tumor-infiltrating IFNγ-producing γδ T cells (52). Similar results were seen in human CRC where a predominately Vδ1^+^ IL-17A-producing γδ T cell population positively correlated with a more advanced tumor stage. This correlation was attributed to an inflammatory DC - γδ17 T cell - MDSC regulatory axis (29). Interestingly, tissue resident memory Vγ2^+^ T cells were also seen in a metastatic mouse model of breast cancer. These Vγ2^+^ T cells produced IL-17A and G-CSF, which promoted the establishment of immunosuppressive intratumoral MDSC (70). Collectively, these studies implicate tissue resident Vδ1^+^ and Vγ2^+^ T cells as tumor growth promoting through IL-17A-mediated MDSC recruitment and immunosuppression in cancer. More importantly, these findings segregate deleterious γδ T cell responses from those which may have a beneficial outcome.

On the other hand, Vδ2^−^ γδ T cells are not limited to pro-tumor effects and effort has been invested into their therapeutic benefits. Intrahepatic Vδ1^+^ and Vδ3^+^ T cells express a CD45RA^+^ CD27^−^ and CD45RA^−^ CD27^−^ phenotype that is nearly absent from the blood. Intrahepatic CD45RA^−^ CD27^−^ Vδ1^+^ and Vδ3^+^ T cells were competent producers of IFNγ and TNFα and also expressed receptors for early activation and tissue retention, such as CD69, that have also been noted in both liver resident NK and CD8^+^ αβ T cell populations (120, 277). CMV infection has notably been one of the drivers of hepatic Vδ2^−^ γδ T cell expansion and memory formation, and these factors appear to have a protective effect against tumor formation. CMV-seropositive patients (infected pre- or post-transplantation) have a reduced risk of skin cancer development and leukemia relapse after kidney or bone marrow transplant, respectively (36, 37). Vδ2^−^ γδ T cells from CMV-infected kidney transplant patients were capable of killing HT29 colon cancer cells *in vitro* (128) and CMV-induced Vδ2^−^ γδ T cells had anti-tumor activity against primary and metastatic tumors in a HT29 xenograft mouse model (278, 279). The characterization of the antigenic specificity of one highly expanded γδ T cell clone from a CMV-seropositive transplant patient revealed that its recognition of stressed (infected or transformed) cells was mediated by the direct binding of the TCR to EPCR, independently of its cargo (33). Similarly, Annexin A2 is upregulated at the surface of stressed cells and can activate another Vδ2^−^ γδ T cell clone (123). However, regardless of which epitope is being recognized, TCR sequencing of intrahepatic Vδ2^−^ γδ T cell populations has revealed that CMV infection can induce expansion, memory phenotypes, and tumor reactivity in a clonally expansive manner (120). Overall, these studies suggest that Vδ2^−^ γδ T cells form T_RM_ cell populations that can clonally expand and cross-react with tumor epitopes to provide anti-tumor immunity.

Knowledge of resident γδ T cell biology is integral for future cancer therapies. Despite intra-tumoral γδ T cell gene signatures being regarded as a favorable prognostic, there is a delicate balance between becoming pro-tumor and anti-tumor γδ T cells (Figure 4). Pro-tumor populations are characterized by γδ17 T cells and their indirect immunosuppressive activity through MDSC (29). On the other hand, anti-tumor populations are characterized by IFNγ producing γδ T cells (52). Notably, IgE response mediated by DETC stress surveillance can have anti-tumor effects (166) as well as potential autoimmune effects (280). A better understanding of how signals in tumor microenvironment shape and potentially polarize γδ T cell cytokine production and signal to other cells would be of great benefit.

## Concluding remarks

The roles of γδ T cells in response to pathogens and commensals and in inflammatory disease and cancer have been an area of expanding interest over the last decade generating significant advances in knowledge. However, our basic understanding of γδ T cell biology is still largely incomplete and lags far behind our understanding of their αβ T cell counterparts, particularly in the area of anamnestic responses. γδ T cells are adapted to their tissue environment which in turn shapes the immune landscape of that environment. Like most cells of the immune system, γδ T cells can appear duplicitous under certain circumstances. On one hand, they can provide beneficial outcomes to the host by conferring anti-pathogen and anti-tumor immunity. On the other hand, they can lead to negative outcomes or exacerbated disease in some inflammatory disorders and cancers. Regardless of their impact, it is now clear that γδ T cell responses encompass both innate inflammatory responses and more traditional adaptive memory responses that provide substantial opportunities for therapeutic targeting. Memory γδ T cell responses may advance a new arm of rationale vaccine design that has broad implications for boosting anti-pathogen or anti-tumor immunity. Vaccines that elicit broadly reactive long-lived circulating or tissue-resident memory γδ T cells may provide protection against a wide range of cancers and infections. Similarly, innate inflammatory or adaptive effector responses may be targeted to enhanced therapeutic modalities with far ranging implications. In the context of a detrimental impact on human health, γδ T cell responses may be blunted or, in the context of cancer, diverted to a lineage that promotes tumor eradication. Thus, memory and tissue-resident γδ T cells represent a lineage of the adaptive immune system that necessitate greater understanding to facilitate the generation of novel therapeutics to promote human health and reduce disease.

## Author contributions

CK wrote the first draft of the manuscript. THC wrote sections of the manuscript. All authors contributed to manuscript revision, read and approved the submitted version.

### Conflict of interest statement

The authors declare that the research was conducted in the absence of any commercial or financial relationships that could be construed as a potential conflict of interest.
